# Multi-district coronary tree involvement in a 17-year-old girl with Williams–Beuren syndrome

**DOI:** 10.1186/s40064-015-1231-0

**Published:** 2015-08-20

**Authors:** Tiziana Serena, Enrico Valerio, Biagio Castaldi, Elena Reffo, Ornella Milanesi

**Affiliations:** Pediatric Cardiology Unit, Department of Woman and Child Health, Medical School, University of Padua, Via Giustiniani, 3, 35128 Padua, Italy

**Keywords:** Williams–Beuren syndrome, Myocardial infarction, Supravalvular aortic stenosis, Multi-district coronary artery disease, Pediatric cardiology

## Abstract

We describe a case of 17-year-old Chinese girl referred to our Pediatric Cardiology Unit for asthenia, reduced exercise tolerance, and dyspnea. Past medical history was relevant for multiple chest pain episodes in childhood and several syncopal episodes, for which the patient had been never evaluated. Clinical examination, electrocardiogram, and echocardiography were compatible with Williams–Beuren syndrome; such condition was later confirmed by genetic analysis. Cardiac magnetic resonance imaging showed transmural fibrosis of the apex with impaired left ventricular ejection fraction (29 %), severe stenosis of aortic sinotubular junction with left and right coronary ostia involvement; more importantly, the whole coronary artery tree beyond ostia was affected by multiple stenosis and aneurysmatic tracts. Ascending aorta proved hypoplastic, with post-stenotic dilation and multiple aneurysms. At the end of the diagnostic process, surgical risk was considered too high to proceed with the correction. The presented case is of educational value since it provides good iconographical illustration of diffuse, multiple-site coronary artery tree involvement, a rather rare co-morbidity in Williams–Beuren syndrome.

**Electronic supplementary material:**

The online version of this article (doi:10.1186/s40064-015-1231-0) contains supplementary material, which is available to authorized users.

## Case report

A 17-year-old Chinese girl was referred to our Pediatric Cardiology Unit for asthenia, impaired exercise tolerance, and dyspnea. Past medical history was relevant for multiple chest pain episodes in childhood and several syncopal episodes, for which the patient had been never evaluated.

Clinical examination showed typical features of Williams Beuren Syndrome (WBS), an harsh (4/6 of Levine scale) systolic murmur at the right upper sterna border, and hyposphagmic peripheral pulses.

Electrocardiogram (ECG) showed left ventricular hypertrophy with diffuse repolarization abnormalities.

Echocardiography detected a severe left ventricular medio-basal hypertrophy associated with significant mid-ventricular pressure gradient (35–40 mmHg), a reduced apical wall thickness (Additional file 1: Video S1), apical dyskinesia (Additional file 2: Video S2), a severe supravalvular aortic stenosis (SVAS) involving coronary ostia, and a mild pericardial effusion. Estimated right ventricular pressure was 50 mmHg.

**Fig. 1:**
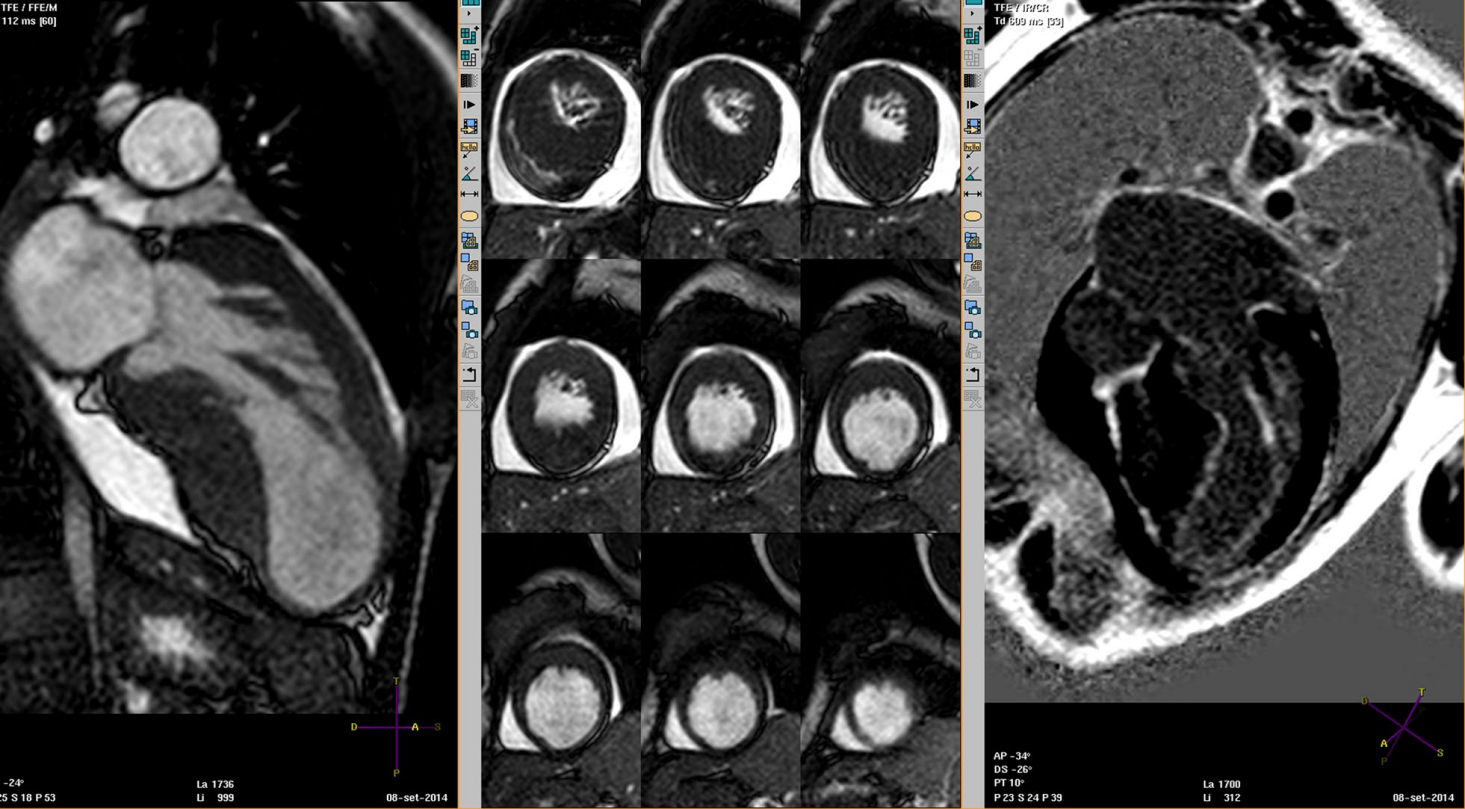
Heart MRI scans showing thinning and aneurysmatic dilation of apical myocardium, associated with transmural myocardial fibrosis

**Fig. 2:**
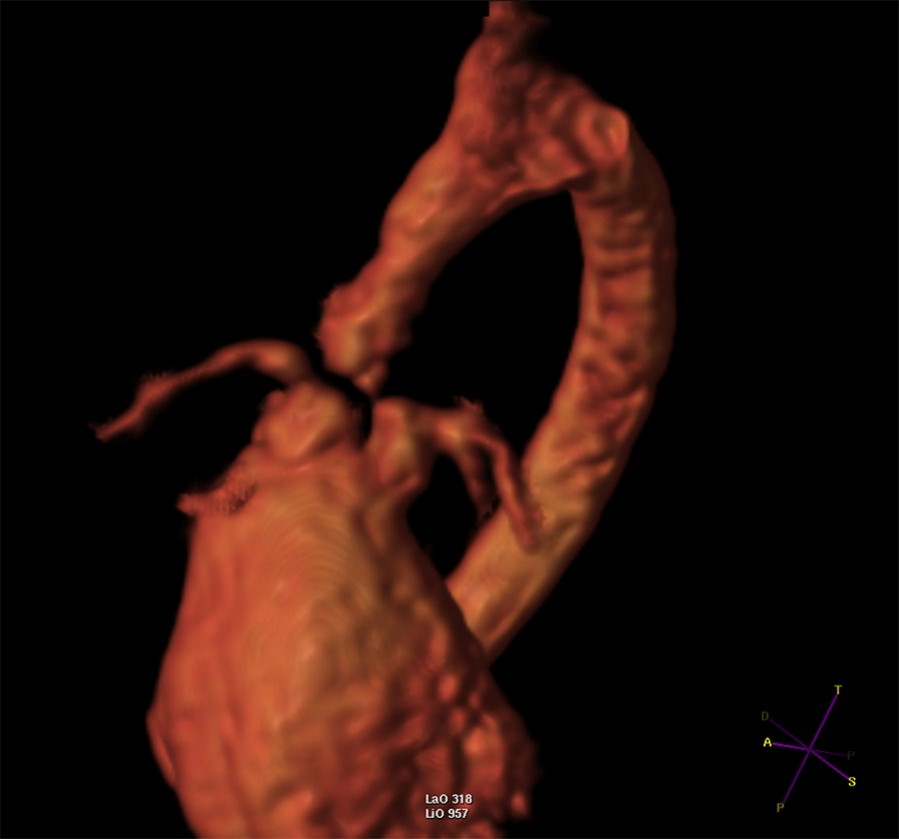
Heart MRI scan showing multiple stenotic and aneurysmatic tracts along the whole coronary artery tree

**Fig. 3:**
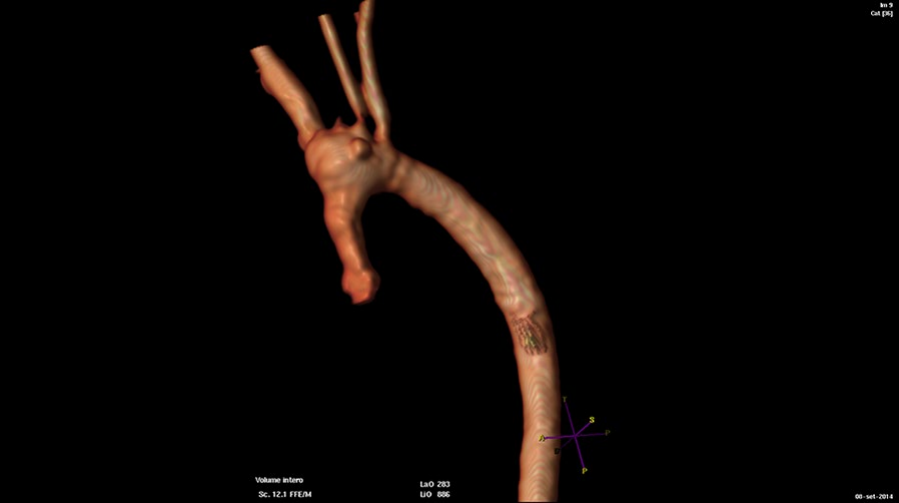
MRI 3D aortic arch reconstruction showing severe hypoplasia and multiple aneurysms of ascending aorta, extending till first brachiocephalic artery; sinotubular junction is also severely stenotic

Cardiac magnetic resonance imaging (MRI) confirmed transmural fibrosis of the apex (Fig. 1); left ventricular ejection fraction (LVEF) was 29 %. Aortic sinotubular junction was severely stenotic, also with involvement of left and right coronary ostia; beyond ostial region, the whole coronary artery tree was affected by multiple stenosis and aneurysmatic tracts (Fig. 2). Ascending aorta proved hypoplastic; aortic arch was affected by post-stenotic dilation and multiple aneurysms (Fig. 3). On the contrary, pulmonary artery tree showed no significant abnormalities.

Fluorescence in situ hybridization (FISH) was performed and documented microdeletion of 7q11.2 region, consistent with WBS.

At the end of the diagnostic process, the surgical plan included aortic valve replacement and annulus enlargement by Konno procedure (aortic annulus 14 mm), SVAS repair including coronary ostia, aortic arch repair including carotid arteries and three coronary artery bypass grafts. However, surgical mortality calculated with logistic euroSCORE tool (Velicki et al. 2014) was 45.7 %, considered too high to proceed with the correction.

Considered the low ejection fraction, the presence of a large apical scar and the mid-ventricular pressure gradient Carvedilol 6.25 mg twice daily was prescribed.

Six months later, the girl was clinically stable and without worsening angina, later she was lost to follow-up.

The patient’s parents gave the written consent to use her data for scientific purposes.

## Discussion

Williams–Beuren syndrome is a rare genetic disorder, characterized by a typical facial dysmorphism, short stature, connective tissue abnormalities (including multi-district vascular disease), infantile hypercalcemia, mental and social disabilities. Vascular issues are mainly due to elastin gene mutation (Morris 2010). Most common heart condition associated to WBS is SVAS (Eronem et al. 2002).

Diffuse SVAS, younger age at correction (<2 years) and post-surgery residual aortic gradient and/or aortic valve disease are associated to poorer outcome (Deo et al. 2012; van Son et al. 1994).

Although aortoplasty is most often performed during pediatric age, deferred SVAS surgery to adult age has proven effective too (Coskun et al. 2007), the main predictor of late death being association with aortic valve disease.

Surgical outcome is heavily influenced by the presence of coronary tree anomalies (Eronem et al. 2002; Imamura et al. 2010; Hornik et al. 2015). As a consequence, preoperative coronary tree evaluation is mandatory in all patients with WBS (Imamura et al. 2010).

Which represents the best technique (cardiac catheterization, cardiac MRI, multi-slice computed tomographic angiography) to perform coronary artery tree evaluation in such patients is still controversial (Imamura et al. 2010).

In our patient, the risk of catheterization-associated mortality due to intraprocedural fatal arrhythmias and/or cardiac arrest was esteemed too high, also considering poor patient compliance which would have needed deep sedation to accomplish the procedure (Krous et al. 2008; Bennett et al. 2005). For such reason, a non-invasive imaging technique was chosen to assess coronary tree anatomy, avoiding sedation or anesthesia. Cardiac MRI was preferred because of its capacity to supply additional information about myocardial fibrosis, left and right ventricular functional and volumetric evaluation, as well as aortic and pulmonary arteries morphology.

The presented case is of educational value since it was associated with diffuse, multiple-site coronary artery tree involvement, a rather rare co-morbidity in WBS. Diffuse coronary artery tree disease was likely responsible for patient’s massive apical myocardial infarction associated with severe depression of left ventricular function. Left ventricular outflow tract was also affected, with severe hypoplasia of aortic annulus and ascending aorta, up to left carotid artery root (see Fig. 2).

## Conclusions

SVAS surgical correction with aortoplasty in WBS patients is feasible with acceptably low mortality and good outcome when performed on time and in absence of distal coronary artery tree involvement (Eronem et al. 2002; Coskun et al. 2007). Coronary ostial stenosis in WBS patients is usually due to SVAS and aortic wall fibrosis at the sino-tubular junction.

In our patient, multiple-site coronary artery tree involvement was detected, a rather unusual co-morbidity in WBS; surgical management of such condition would have required multiple coronary bypass grafts (CABG). The needing of potentially multiple CABG, extensive aortoplasty, and carotid artery patch plasty, in the setting of a severely impaired left ventricular systo-diastolic function and reduced LVEF made surgical risk unacceptably high.

## Additional files

Additional file 1:
**Video S1.** Left parasternal long axis view showing severe left ventricular medio-basal hypertrophy with reduced apical wall thickness. Additional file 2:
**Video S2.** Left parasternal apical short axis view showing apical dyskinesia with “twisting” motion.

## Abbreviations

ECG: electrocardiogram
WBS: Williams–Beuren syndrome
MRI: magnetic resonance imaging
LVEF: left ventricular ejection fraction
FISH: fluorescence in situ hybridization
SVAS: supravalvular aortic stenosis
CABG: coronary bypass grafts
